# Intersubjectivity and the Emergence of Words

**DOI:** 10.3389/fpsyg.2022.693139

**Published:** 2022-04-26

**Authors:** Herbert S. Terrace, Ann E. Bigelow, Beatrice Beebe

**Affiliations:** ^1^Departments of Psychology and Psychiatry, Columbia University, New York, NY, United States; ^2^Department of Psychology, St. Francis Xavier University, Antigonish, NS, Canada; ^3^New York State Psychiatric Institute, Columbia University Medical Center, New York City, NY, United States

**Keywords:** intersubjectivity, bi-directional communication, dyadic relationship, triadic relationship, contingency, joint attention, words

## Abstract

Intersubjectivity refers to two non-verbal intersubjective relations infants experience during their first year that are precursors to the emergence of words. Trevarthen, a pioneer in the study of intersubjectivity, referred to those relations as primary and secondary intersubjectivity. The former, a dyadic coordination between the infant and her caregiver, begins at birth. The latter, a triadic coordination that develops around 9 months, allows the infant and a caregiver to share attention to particular features of the environment. Secondary intersubjectivity is crucial for an infant’s ability to begin to produce words, at around 12 months. Much research on the social and cognitive origins of language has focused on secondary intersubjectivity. That is unfortunate because it neglects the fact that secondary intersubjectivity and the emergence of words are built on a foundation of primary intersubjectivity. It also ignores the evolutionary origins of intersubjectivity and its uniquely human status. That unique status explains why only humans learn words. This article seeks to address these issues by relating the literature on primary intersubjectivity, particularly research on bi-directional and contingent communication between infants and mothers, to joint attention and ultimately to words. In that context, we also discuss Hrdy’s hypothesis about the influence of alloparents on the evolution of intersubjectivity.

## Introduction

“Before language, there was something else more basic, in a way more primitive…that propelled us *into* language…that something else was *social engagement with each other*. The links that can join one person’s mind with the mind of someone else—especially, to begin with, emotional links—are the very links that draw us into thought…The foundations of language were laid at the point when ancestral primates began to connect with each other emotionally in the same way that human babies connect with their caregivers” (Hobson, 2002, p. 2 italics in original).

Social and emotional non-verbal engagement between an infant and her caregiver are, as noted in the epigraph, crucial for the growth of language. These early forms of engagement are precursors of an infant’s first words and are referred to as intersubjectivity, the focus of this article. Our goal is to show why intersubjectivity is necessary for an infant’s acquisition of words and for the emergence of words in our evolutionary history.

The evolution of language has been described as “the hardest problem of science” (Christiansen and Kirby, 2003). That is because many scholars have regarded language as a singular event. As such, the theory of evolution cannot explain it.

At the very least, language consists of words and grammar. Here, we are concerned with the emergence of words, rather than grammar, because words emerge before grammar, both phylogenetically and ontogenetically (Studdert-Kennedy and Terrace, 2017). We argue that the social foundations for the emergence of words provide a partial, but nevertheless important, answer to the hardest problem.

### How Does Intersubjectivity Lead to Words?

Trevarthen, the premier theoretician of intersubjectivity, argued that words emerge at the end of the first year because of the cumulative effect of the two stages of intersubjectivity: primary and secondary. Primary intersubjectivity refers to reciprocal emotional and attentional coordination between an infant and a caregiver during face-to-face interaction, a dyadic relation that begins at birth. Secondary intersubjectivity, which typically begins toward the end of the first year, refers to a triadic relation between an infant, her caregiver, and nearby objects to which they jointly attend. It is based on the cooperative exchange of referential gestures between an infant and her caregiver (Trevarthen and Hubley, 1978; Hubley and Trevarthen, 1979).

The production of words, at about 12 months, is a crowning achievement of secondary intersubjectivity. Unfortunately, that achievement led many psychologists interested in the origin of words to focus more on secondary than on primary intersubjectivity (e.g., Bates et al., 1979; Nelson, 1996a,b; Tomasello, 1999). It not only implies a discontinuity in the development of intersubjectivity, but it also overlooks the fact that *secondary intersubjectivity could not emerge without primary intersubjectivity*. Emotional and attentional sharing are needed for the acquisition of words.

We agree with Trevarthen’s view that progress toward the emergence of words is gradual, that it begins at birth, and that it encompasses both primary and secondary intersubjectivity. Here, we review recent studies that describe the nature of this development and the continuity of primary and secondary intersubjectivity. We also note that much additional work remains to be done.

How does the emergence of words in our evolutionary history inform our understanding of the development of words? As noted earlier, the theory of evolution cannot explain the origin of language as a singular event. Intersubjectivity is a missing link. Although animals can perceive emotions in others, they are limited in their ability to share them. Intersubjectivity allows that to happen, first by sharing emotion and attention dyadically, then by sharing attention to objects and, ultimately, by the exchange of words.

We end the article with a discussion of why intersubjectivity became crucial for the emergence of words in our evolutionary history. In that context, we describe Hrdy’s theory of how intersubjectivity evolved from the practice of collective breeding by recent ancestors (Hrdy, 2009; Hrdy and Burkart, 2020). While discussing the emergence of words, we define them in a way that not only distinguishes them from the signals that animals use to communicate, but also shows why they are uniquely human.

We begin by describing basic features of primary and secondary intersubjectivity, as defined by Trevarthen, and more recent developments, such as “protophones,” a precursor of babbling, that has some of the functional properties of words. We return to protophones at the end of the article to note that they may have played a prominent role in the evolution of words (Oller and Griebel, 2021).

## Primary Intersubjectivity

Primary intersubjectivity is based on an infant’s innate ability to coordinate gaze, vocalization, facial expression, and gesture with those of a parent. Such coordination is identified through correspondences in the form, timing, and intensity of these behaviors, and the contingencies (predictable sequences) that organize these exchanges.

Trevarthen discussed many examples of dyadic communication between an infant and her caretaker as instances of primary intersubjectivity. As opposed to experimental paradigms, such as imitation, those examples were drawn from observations of quasi-naturalistic, ongoing face-to-face communication. This article limits itself to such studies.

To study primary intersubjectivity, Trevarthen and subsequent researchers videotaped mothers and infants, seated face-to-face, using two cameras, one aimed at the mother, the other at the infant, generating a split-screen view (Stern, 1971; Brazelton et al., 1974; Trevarthen, 1977, 1980). Mothers were instructed to play with their infants as they would at home. Researchers could then rate the behavior of mothers and infants for variables, such as gaze direction, facial expression, vocal affect, head orientation, and touch.

The method of microanalysis was used to analyze such interactions. Beebe (2014, p. 4) described how microanalysis reveals coordination between an infant and her mother that is “so rapid and subtle that they are not quite grasped in real-time. By slowing down the movements, frame-by-frame microanalysis identifies remarkably beautiful moments, such as both partners rising up…into glorious sunbursts of smiles. It also reveals very disturbing moments, such as maternal anger or disgust faces, or infants becoming frantically distressed or frozen in alarm.” The tiny behaviors revealed by microanalysis, such as rapid shifts of gaze, head, hand, mouth-opening and closing, are often as short as 250 ms (Beebe, 1982).

### The Newborn’s Preparedness for Primary Intersubjectivity

Infants are born prepared to engage in primary intersubjectivity. Evidence to support that view comes from an infant’s sensitivity and responsiveness to a caregiver’s voice, facial expressions, and gestures. Sensitivity to a mother’s voice is actually present prior to birth. The fetus can recognize the mother’s voice and can respond to auditory stimuli from as early as the 26th week of gestation (Eisenberg, 1976). Components of speech, such as pitch, rhythm, stress, and some phonetic information, can be transmitted through the uterus (DeCasper and Fifer, 1980; Querleu et al., 1988; Lecanuet and Granier-Deferre, 1993).

Prenatal exposure to the mother’s voice has been shown to affect postnatal auditory preferences. At birth, newborn infants prefer to listen to their mothers’ voice (DeCasper and Fifer, 1980; Fifer and Moon, 1989) and can recognize speech samples from stories read to them prenatally by their mothers (Decasper and Spence, 1986). Neural evidence suggests that prenatal experience with language configures the neonate’s brain to be responsive to the language heard prior to birth (May et al., 2011).

### Protoconversation

**Figure 1:**
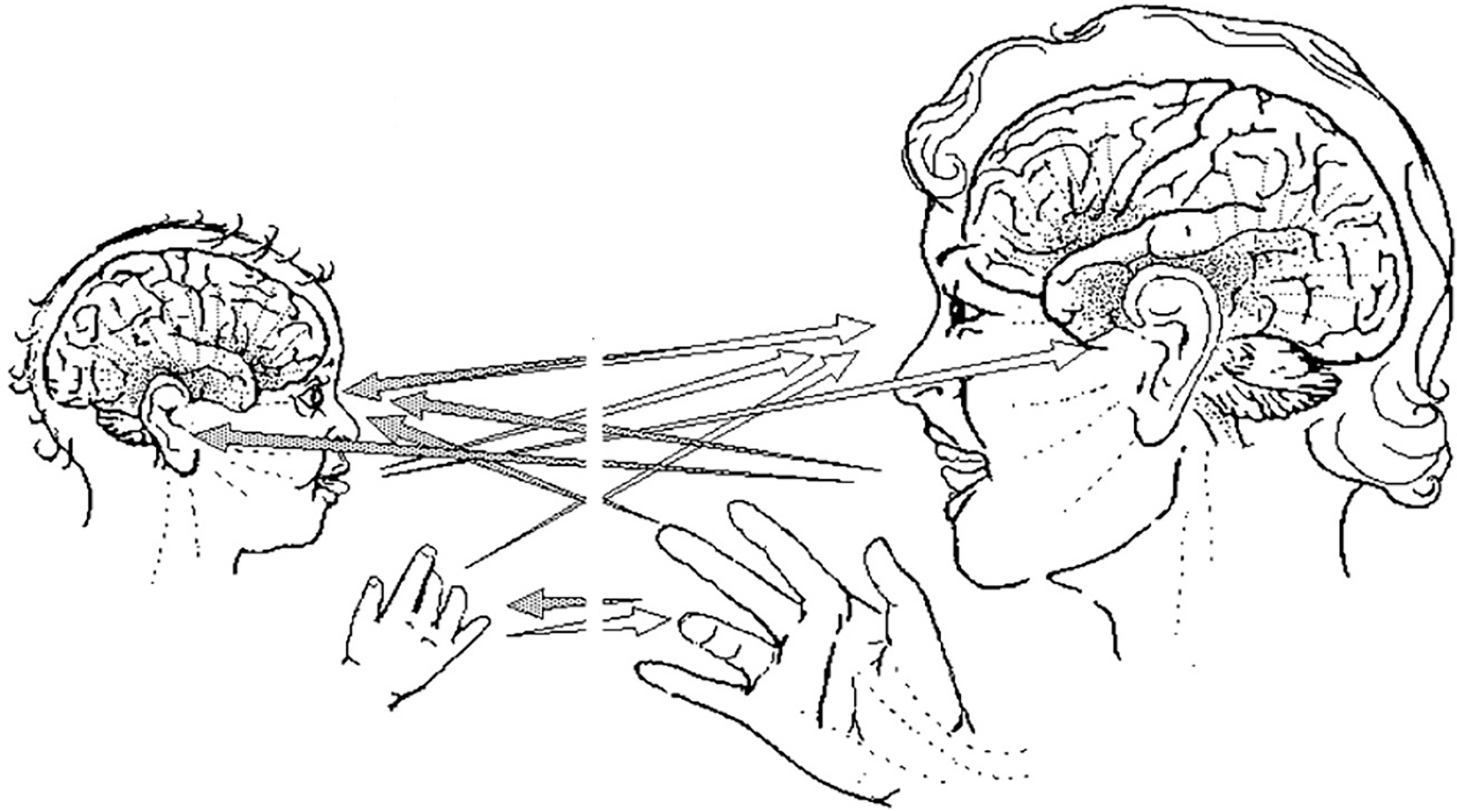
Illustration of multiple channels of protoconversation between an infant and her mother (Trevarthen et al., 2006, Figure 12).

Infants and caregivers engage in dyadic exchanges of attention, vocalization, and facial expression. These exchanges are referred to as protoconversations. The scope of protoconversations is illustrated in Figure 1, which shows some of the channels of communication between an infant and her mother. Trevarthen commented that: “…*subtle timing and complementary emotional expressions in protoconversations by 2-3-month-olds was perceived to be preparatory to linguistic communication*… they achieve their meaning…by exercise of non-linguistic forms of facial, vocal, and gestural expression and interaction with partners” (Trevarthen, 1998, p. 18, italics in original).

Trevarthen (1979) credits Bateson (1979) with the discovery of protoconversation. She described an important turn-taking relation between the utterances of 2-month-old infants and their mothers. In response to the mother saying for example, “What you going to say?,” “Huh?,” “Oh my!,” “You going to be a good boy today?,” the infant often responded by cooing, grunting, whimpering, and making other infant sounds (Bateson, 1979, p. 104). The onsets of the mother’s comments and the infant’s responses were strongly correlated. Because there was little temporal overlap between those utterances, Bateson referred to them as “protoconversations.” Infants and mothers frequently alternate their utterances, just as adults do when they use language to converse.

Stern et al. (1975) argued that caregivers of 3–4-month-old infants have two modes of vocal interaction: simultaneous and turn-taking. Stern et al. (1975) termed these modes “coaction” and “alternation.” In coaction, an infant and her caregiver’s vocalizations overlap, as for example, when the infant cries and the caregiver attempts to soothe the infant vocally. In alternation, infant and caregiver take turns, as in protoconversation. Malloch and Trevarthen (2009) identified a narrative structure within these protoconversations, evident in the timing and reciprocity of the gestures and utterances (Delafield-Butt and Trevarthen, 2015), a structure that Dan Stern first described as “proto-narrative” (Stern, 2000).

#### Protophones Within Protoconversation

In a groundbreaking research program, Oller (2000) showed that protoconversations actually begin shortly after birth. Infants engage vocally with their caretakers by uttering “protophones,” primitive precursors of speech that consist of squeals, growls, and vowel-like vocalizations, called *vocants*. Squeals are vocalizations that are of a “notably higher than normal range of the infant”; growls, “notably lower than the normal range,” and vocants, “in the mid pitch range of the infant” (Oller et al., 2013, supplement, p. 19).

A remarkable feature of protophones is their dual function. In addition to their use in protoconversation, they often occur endogenously, not directed at anyone (Long et al., 2020; Oller et al., 2021). Only humans, among primates, have been shown to produce endogenous vocalizations (Oller et al., 2019).

Infants need no encouragement to vocalize. Indeed, they seem to produce protophones to explore sound with no purpose other than to hear their own voice. Protophones therefore form the foundation of infants’ vocal interactions. In their use in protoconversations, protophones provide one of the most important channels of primary intersubjectivity.

Oller distinguished protophones from cries, laughter, and vegetative sounds (coughs, sneezes, burps, etc.) because the functions and affective states of the latter utterances are fixed and are shared with other species. In contrast, protophones have “functional flexibility” in that they can be used in any affective state. This functional flexibility allows protophones to play an important role in language development. Like words, protophones do not have species-specific meaning: “Early protophones have a special role in language development and evolution because they are the first sounds to be free of specific fixed functions and thus reveal…the flexibility required for language” (Oller et al., 2013, p. 6322).

Functionally flexible protophones can express “positive, negative, and neutral emotional states on different occasions” (Oller et al., 2013, p. 6318). After an infant utters a protophone, her caretaker’s response is based on intuitive judgments of the infant’s affect while producing that protophone. In response to an infant’s protophone, such as a squeal, a caregiver might respond with positive affect if the squeal was accompanied by positive affect. When the same sound is expressed with neutral affect, the caretaker might respond in kind. If the squeal is expressed with negative affect, the caretaker might vocalize with a sympathetic sound.

Such observations suggest that protophones can be detached from any particular emotional state, similar to the way that words can be used to represent different emotional states. That type of flexibility has not been reported in non-human primate vocalizations.

From birth, protophones occur at substantially higher frequencies than stereotyped species-specific vocalizations, such as cries (Oller et al., 2013). Yoo et al. (2018) were the first to investigate the temporal relation between an infant’s protophones and cries, and a caregiver’s vocal response. Even during the infant’s first 3 months, caregivers were likely to take turns interacting with protophones, but not with cries. When an infant produced protophones, mothers often responded in a protoconversational manner.

#### Turn-Taking and Protoconversation

Vocal turn-taking provides a key pattern of interaction that organizes exchanges during primary intersubjectivity. Turn-taking is not, however, unique to humans. Members of many non-human species take turns while interacting with one another (Pika et al., 2018). Examples can be found in all major branches of primates (Levinson, 2016), in non-primate mammals [whales (Miller et al., 2004; Schulz et al., 2008; Morisaka et al., 2013), dolphins (Lilly, 1962; Nakahara and Miyazaki, 2011), bats (Carter et al., 2009), and elephants (Leighty et al., 2008)], in more than 100 different species of birds (Dahlin and Benedict, 2014), and even in insects (Mason, 2009).

In these species, the functions of turn-taking include mutual recognition, maintenance of contact between partners, mutual defense of territories, reproductive synchrony, and mate location. In many instances, the structure of turn-taking is similar to that of humans. Turns are relatively short (from less than a second to a few seconds) and the gap between turns is brief (often as little as 200 ms). Similarity in the form and structure of turn-taking in non-human species and humans notwithstanding, there are fundamental differences in content and modality.

Regarding content, turn-taking responses in non-human species are fixed in that they vary little over successive turns. In humans, the content is arbitrary, that is, variable and flexible. Evidence can be found in vocal exchanges between infants as young as 2 months and their caregivers (e.g., Bateson, 1979). As noted in the previous section, an infant’s affect varies in such exchanges. At 3 months, the quality of the infant’s utterances varies as a function of whether she is responding contingently to her mother’s vocalizations (Bloom et al., 1987; Gratier et al., 2015).

Regarding modality, most studies of turn-taking in humans focus on vocalization or speech. It has recently been shown, however, that turn-taking occurs in exchanges of sign language (de Vos et al., 2015). That suggests that spoken and sign languages follow similar time courses in the planning and production of conversational utterances. The multiple modalities of gesture and voice produce what Trevarthen and Delafield-Butt (2013) identify as the origin of an invariant “narrative” form in pre-verbal protoconversation.

### Babbling and Phonetic Perception

Canonical babbling begins at about 6 months (Vihman, 2014), and may originate in infants’ endogenous vocal exploratory activity. It is characterized by syllables with at least one vowel-like element and one consonant-like element, with a rapid, adult-like transition between consonant and vowel [phonetical representation: for example, (ba), (di), and (da)]. The rapid transition between consonants and vowels is a defining feature of the difference between pre-canonical and canonical syllable productions.

Mother–infant interactions are the prominent social context influencing infants’ canonical babbling. Goldstein and Schwade (2008) showed that 9-month-old infants modify their canonical babbling in response to their mothers’ contingent utterances. Under a contingent condition, mothers were asked to respond to their infants’ babbling either by speaking a resonant vowel or by speaking a word that alternated a consonant and a vowel. Under the non-contingent condition, recordings of the mother’s responses were not synchronized with the infant’s babbling.

Infants given contingent feedback restructured their babbling by incorporating patterns of their mother’s speech. Infants given non-contingent feedback did not incorporate patterns of their mothers’ speech.

Infants hearing contingent resonant vowel responses increased their resonant vowels. Similarly, infants hearing contingent words with consonant–vowel sounds increased the frequency of their consonant–vowel syllables. Although the sounds the infants produced were likely already in their repertoire, there was an overall increase in the frequency of particular phonemes. These phonemes reflected the mothers’ patterns of speech. In this manner, maternal speech influences infants’ canonical babbling, an important step in word learning.

Related research provides evidence of phonetic perception. Unlike adults, young infants readily discern phonetic properties used in languages to which they have not been exposed (Eimas et al., 1971). But this ability declines sharply between 6 and 12 months of age (Werker and Tees, 1984). Kuhl et al. (2003) exposed 9-month-old English-learning infants to Mandarin in 12 lab sessions. The infants exposed to Mandarin continued to perceive the phonetic properties of Mandarin, but that ability declined in control infants. However, the ability to perceive the phonetic properties of Mandarin was found only if the exposure was from live interactions between Mandarin speakers and the infants, rather than from video or audio-only exposure to the same Mandarin speakers. Similarly, when 9-month-old English-learning infants were exposed to 12 sessions with Spanish speakers in live interactions with toys, infants’ social engagement with the Spanish speakers predicted their phonetic discrimination of Spanish (Conboy et al., 2015). As noted by Kuhl et al. (2003), an infant’s ability to neurally code the phonetic properties of language interacts with the social context in which language is heard.

## Secondary Intersubjectivity

Secondary intersubjectivity generally emerges between 9 and 12 months and includes joint attention. Joint attention refers to the *triadic* coordination of an infant and her caregiver with objects or events in the immediate environment. It is based on sharing one’s attention, feelings, and intentions with regard to external objects (Trevarthen and Hubley, 1978; Trevarthen, 1993). As we argue below, joint attention is crucial for the production of an infant’s first words.

The transition from dyadic forms of shared attention and emotion during face-to-face interaction to triadic forms of shared attention is one of the most dramatic developments during an infant’s first year. Whereas shared attention (parallel looking) is not uniquely human, joint attention is (Tomasello, 1999; Tomasello et al., 2005; Zlatev, 2008). For example, when two chimpanzees orient to the same object, or when one chimpanzee follows another’s gaze, they share attention to that object.

What is missing in this and in other examples of shared attention is visual and/or emotional acknowledgment that they each see the same object. Consider an infant who points to an object to which her caregiver is attending, and then gazes at her caregiver. That is evidence of what Bruner (1975) described as a “meeting of the minds,” or what Tomasello (1995) subsequently referred to as “knowing together.”

### Joint Attention

**Figure 2:**
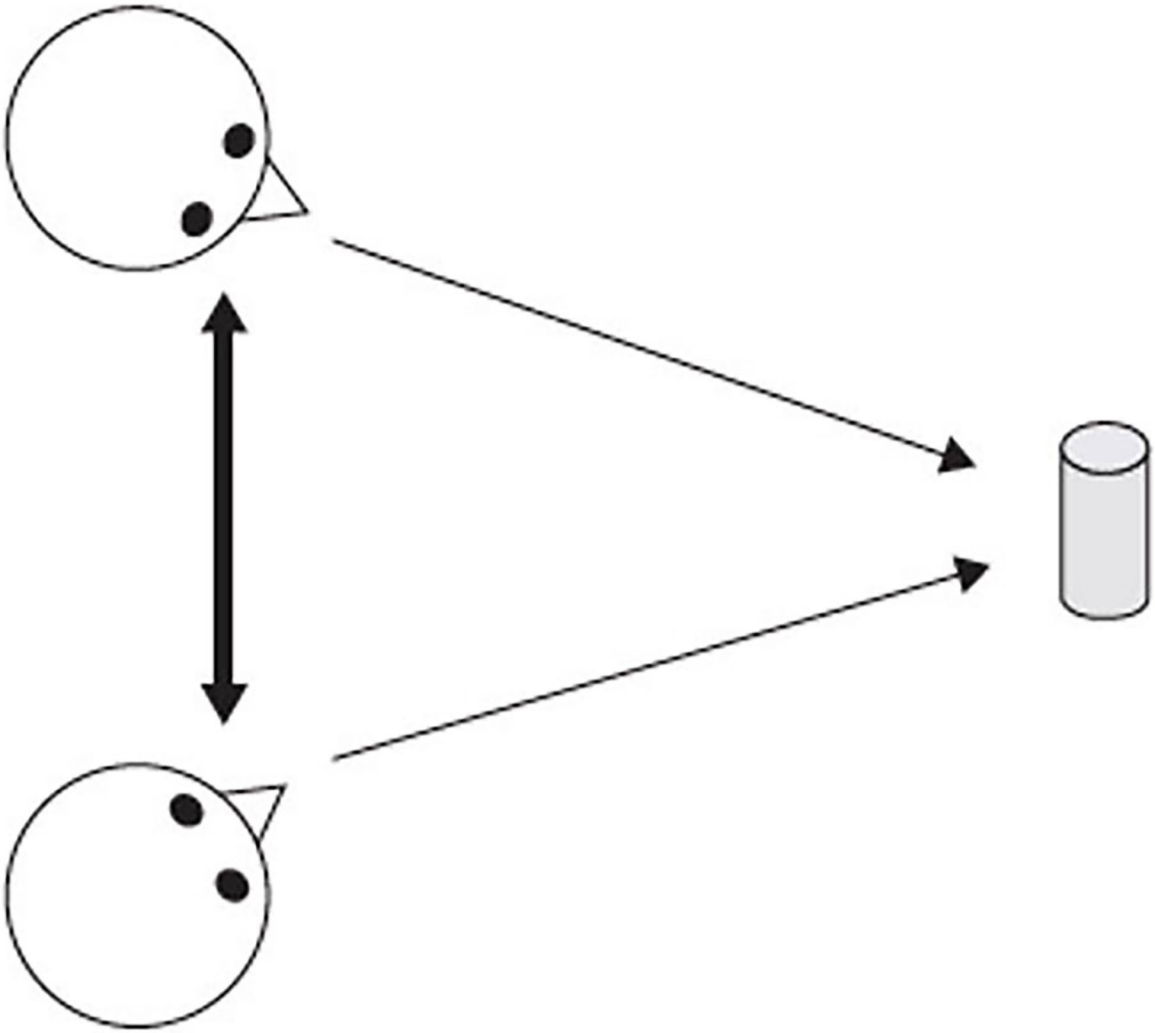
The joint attention triangle. The bold arrow represents how two individuals “know together” that they are sharing attention to the same object. Adapted from Figure 2.1 of Carpenter and Call (2013).

Shared attention and reciprocal acknowledgment of such attention are necessary for the establishment of joint attention. The difference between these phenomena is shown in Figure 2.

What makes attention *joint* is shared attention to an object that includes reciprocal acknowledgment of that sharing (Carpenter and Call, 2013). The thin arrows in Figure 2 illustrate shared attention (parallel looking). The bold arrow represents bi-directional sharing, some form of social behavior, for example, looking, smiling, vocalizing, that acknowledges that each individual knows that they are both looking at the same object.

Joint attention is critical to our argument that early intersubjectivity contributes to the emergence of words because joint attention predicts subsequent language outcomes, for example, the age at which words are first produced and vocabulary size (Tomasello and Todd, 1983; Tomasello et al., 1986; Tamis-LeMonda et al., 1996; Carpenter and Call, 2013).

### The Continuity vs. Discontinuity Debate Between Primary and Secondary Intersubjectivity

Many psychologists interested in the origins of words have ignored the contribution of primary intersubjectivity to the emergence of secondary intersubjectivity that culminates in word learning (e.g., Bates et al., 1979; Nelson, 1996a,b; Tomasello, 1999). For example, Tomasello (1999) has been a strong advocate of a discontinuity between primary and secondary intersubjectivity. As evidence, he cites a “9-month cognitive revolution” in which infants acquire shared intentionality, the motivation to share attention with others (see Racine et al., 2014). That includes the ability to perceive that another is attending to the same object as the self. The 9-month revolution is based on experiments on imitative learning, social referencing, goal detection, and other joint attentional capacities that emerge between 9 and 12 months (Carpenter et al., 1998). The results of those experiments led Tomasello to reject Trevarthen’s position of strong continuity between primary and secondary intersubjectivity.

Although Tomasello and his colleagues have amassed evidence that 9-month-old infants exhibit shared intentionality, we argue that shared intentionality is built on the foundation of primary intersubjectivity. One cannot share attention triadically until it can be shared dyadically (Oller, 2000; Oller et al., 2016). Longitudinal studies show no evidence that infants begin joint attention before experiencing extensive dyadic interaction (Oller, 2000; Legerstee et al., 2007; Bigelow et al., 2010). Logically and empirically, triadic interactions incorporate dyadic sharing.

The “9-month revolution” is based on a combination of factors that have their origins in primary intersubjectivity. These include the role of early dyadic interactions, mother and infant reciprocal contingent coordination in these early interactions, and how infants interact with objects before 9 months.

## What Is the Empirical Evidence That Primary and Secondary Intersubjectivity Are Continuous?

Commenting on the literature’s disconnect between primary and secondary intersubjectivity, Legerstee et al. (2007, p. 298) provided the following diagnosis:

The problem is that theorists who propose that infants do not engage in triadic engagement until 9 months of age seldom investigate infants below these ages (Tomasello, 1995; Carpenter et al., 1998), whereas those who argue for a relationship between dyadic and triadic communication seldom venture beyond the age of 3 months (Tronick et al., 1978; Tronick, 1981; Murray and Trevarthen, 1985).

There are, however, some suggestions of continuity between primary and secondary intersubjectivity. In what follows, we describe how interactions in *early infancy* relate to joint attention and the production of words toward the end of the first year. We first present evidence that infants engage with mothers around objects earlier than the 9-month revolution that Tomasello proposed.

### Early Mother–Infant Engagement With Objects

Some studies have examined infant–adult triadic engagement with objects under 9 months of age. For example, de Barbaro et al. (2013) measured shifts in mother–infant sensory-motor coordination longitudinally, while infants were looking at or manipulating toys at ages 4, 6, 9, and 12 months. At 4 months, infants attended to a single toy at a time, with mothers engaged in active scaffolding by moving toys toward or away from the infants. At 6 months, infants maintained prolonged attention to their toys, often sharing that attention with their mothers. At 9 months, infants were able to handle two toys simultaneously, and bouts of mother–infant turn-taking occurred around their shared interest in objects. At 12 months, infants often verbalized while watching their mothers and attempted to imitate their mothers’ actions on the toys. At each age, de Barbaro et al. (2013) documented that infants’ actions on toys enhanced those observed earlier, showing continuity in how infants engage with objects. Importantly, infants smiled and gazed at their mothers while playing with toys prior to 9 months.

Grossmann and Johnson (2010) explored the activation of 5-month-old infants’ prefrontal cortex during joint attention with an adult and an object. The prefrontal cortex of the brain is activated during joint attention in adults (Schilbach et al., 2013). At 5 months, infants shared looks to an adult and object. Like adults, the left dorsal prefrontal cortex was activated when they engaged in joint attention. The authors speculated that the human infant is neurobiologically prepared to participate in joint attention and that this ability is available at 5 months.

Striano and Bertin (2005) examined mother–infant and stranger–infant engagement with objects longitudinally at infant ages 5, 7, and 9 months. They showed that infants coordinated attention to an object with mother, and with a stranger, at 5 and 7, as well as 9 months. Triadic coordination of attention with positive affect increased gradually, rather than abruptly, from 5 to 9 months.

The research described in this section on the ways that infants coordinate interest in toys and engagement with their caretakers suggests that Tomasello’s “9-month revolution” is actually an incremental process that begins at 4 months. Infants gradually integrate objects into their dyadic interactions.

## Expanding the Domain of Primary Intersubjectivity

In this section, we describe research on mother–infant interaction in the first few months of life, in particular the importance of contingency in early mother–infant interactions, and how experimental disruptions of contingency can disturb them. We describe research that explores the development of the coordination of face-to-face exchanges across the first few months, especially the salience of bi-directional vocal exchanges. We then consider how early contingent interactions are related to joint attention and the emergence of words.

The insightful descriptions of primary intersubjectivity by Trevarthen (1979) were based mainly on single case or small N studies. Subsequent research with larger samples provided an expanded description of how mothers and infants engage in face-to-face communication during primary intersubjectivity.

Trevarthen argued that primary intersubjectivity was organized by *correspondences and contingencies of behavior* between mother and infant (Beebe et al., 2003). Correspondences include matching of form, timing, and intensity of behaviors, for example, both partners smiling, vocally pausing for similar durations, or both emitting a high-pitched squeal.

### Contingency

Whereas correspondences involve particular behaviors per se, contingency addresses the structure of behavioral sequence across time. *Contingency* refers to sequential constraint: a significant probability that a prior behavior predicts a subsequent behavior. Recent studies on the early development of primary intersubjectivity have focused more on contingency of interactions than on correspondences of form.

In a study of mother–infant face-to-face communication at infant age 4 months, which coded second-by-second behavior from split-screen video and assessed contingency using time-series models, Beebe et al. (2016) showed contingent coordination between mother and infant facial affect, vocal affect, head orientation, and gaze. Contingent coordination was bi-directional, that is, mothers’ behavior affected that of infants, and vice versa. Across the group, in all the modalities assessed, each partner followed the direction of the other’s change.

**Figure 3:**
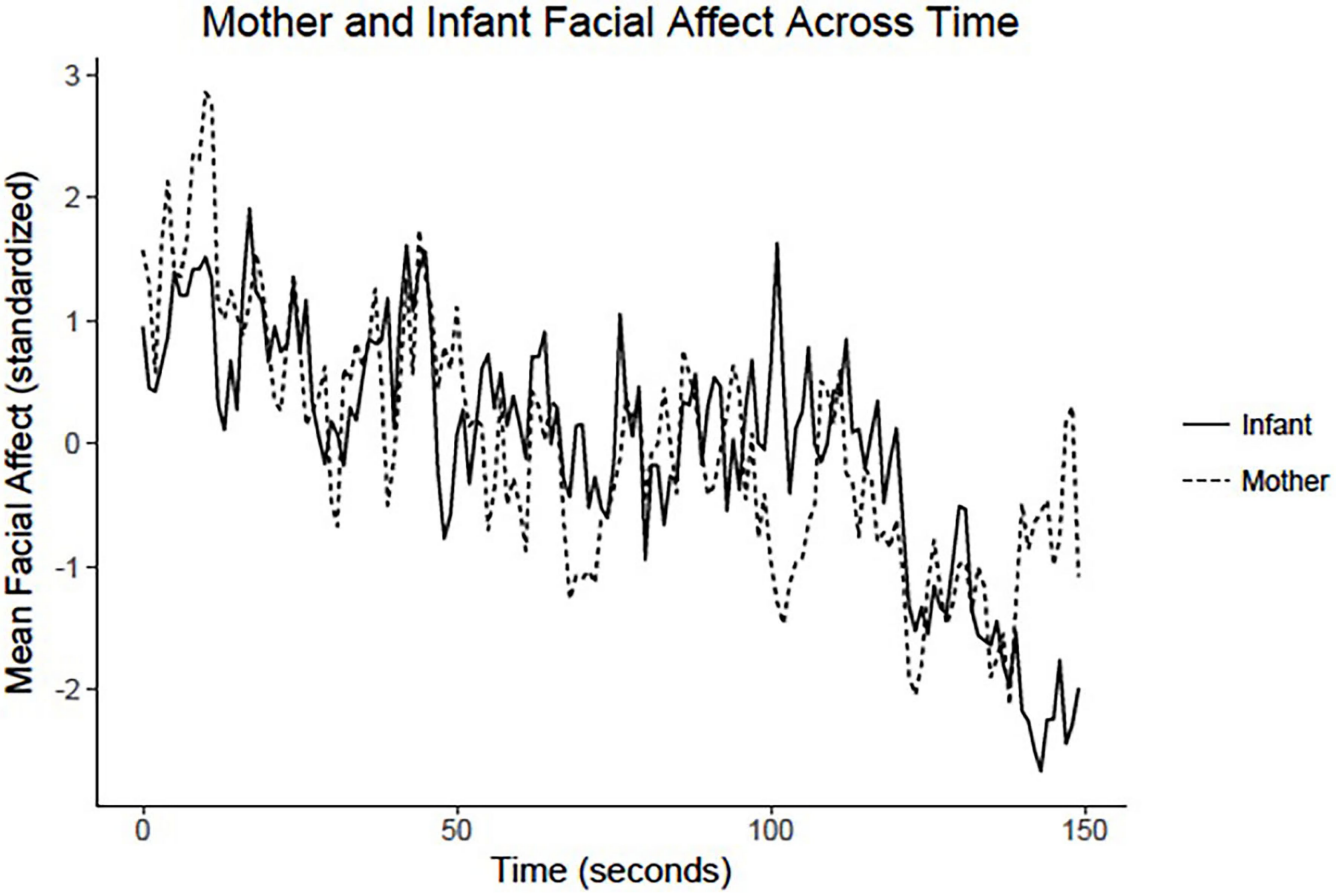
Second-by-second ratings of mother and infant facial affect during sessions (150 s) of mother–infant dyads. This illustration of mothers and infants following the others’ direction of affect change is based on an across-group (*n* = 132) documentation of bi-directional contingent coordination *via* multi-level time-series modeling (Beebe et al., 2016). See text for additional details. Data obtained from Table 1 of Beebe et al. (2016).

Figure 3 shows an example of contingent coordination (Beebe et al., 2016), by depicting second-by-second ratings of mother and infant facial affect during face-to-face interaction. It shows how mothers and infants closely followed each other’s direction of affect change.

Beebe et al. (2016) also showed that this bi-directional process was *asymmetrical*. Mothers coordinated and adjusted their contingent behaviors to their infants more than infants adjusted to their mothers. That asymmetry is important in understanding that the mother has a key role in providing the conditions in which this bi-directional interactive process develops. Maternal contingent responsiveness is important to the infant’s increasing social capacity that will lead to joint attention and words. But despite this asymmetry, infants have a powerful role in these interactions and, ultimately, it is the infant’s contingent vocal response that will lead to the onset of words.

Infants are sensitive to the ways in which their behaviors are responded to contingently by social partners (Murray and Trevarthen, 1985; Tamis-LeMonda et al., 2001). Others’ contingent responsiveness to infant behavior leads infants to expect that they can affect their partner’s behavior through their own actions, enhancing their sense of agency (Tarabulsy et al., 1996; Haith and Benson, 1998; Harrist and Waugh, 2002; Bigelow and Rochat, 2006). Infants are aware of their agency very early, possibly from birth or even earlier, as demonstrated by their actions on their own bodies (Rochat and Striano, 2000) and in the physical environment (Watson, 1979). However, when in interactions with others during primary intersubjectivity, infants’ awareness of their agency increases as they notice the effect of their behavior on others.

#### Disruptions of Contingency

Responses of infants to Still Face and Replay experiments provide further evidence of infant expectancies. Not only are expectancies an important foundation of the infant’s communicative capacity (Fagen et al., 1984; Tronick, 1989; Gros-Louis et al., 2014), but they are also critical in the development of joint attention, which requires the expectation of being able to influence a partner’s attentional focus.

In the Still Face Paradigm, mothers and infants engage in a face-to-face task in three phases (Tronick et al., 1978). Initially, mothers and infants interact as they normally would, providing a baseline. Mothers are then instructed to become completely still-faced, looking at the infant with a neutral expression, without touching or talking. Finally, they resume normal interaction.

If the infant expects the mother to be responsive, the still-face phase should violate that expectation, and the infant should react differently in the still-face phase than in the baseline or resumption of play phases. Such changes are reliably seen from 2 months of age (Mesman et al., 2009). Infants reduce their attention and positive affect when the mother becomes unresponsive during the still-face phase, as compared to the interactive phases.

The Replay Task provides an even more stringent test of the infant’s expectations of contingent responsiveness (Murray and Trevarthen, 1985). Mothers and infants engage over closed-circuit TV, which does not disturb mutual contingent responsivity. First mothers and infants interact as they normally would. Then the infants view a replay of the *previous* interaction, such that the mother’s responsiveness to the infant’s current behavior is absent. By 4 months (Hains and Muir, 1996; Bigelow and Decoste, 2003), and in some studies earlier (Murray and Trevarthen, 1985; Nadel et al., 1999), infants respond to the replay phase much like the still-face phase. These studies show that infants have developed expectations for their mother’s contingent responsiveness, not just expectations for infant-directed facial expressions and vocalizations.

These experimental disruptions of contingency indicate that infants are very sensitive to the contingency structure and that ordinary ongoing infant social behavior is disturbed when contingency is disrupted. The nature of the contingency structure is thus a key aspect of primary intersubjectivity.

### Early Developmental Changes in Mother–Infant Face-to-Face Interaction

Most research on face-to-face communication during the period of primary intersubjectivity has focused on infants at 3–4 months. By that time, mother–infant bi-directional contingent coordination is well-established (Cohn and Tronick, 1988; Beebe et al., 2016). There are, however, important developments prior to this time that allow us to observe the growth of such coordination.

A major shift in infant perceptual-motor abilities occurs around 2 months. These include increases in the infant’s ability to maintain an upright posture, to sustain visual attention, and to explore the internal features of the partner’s face. Such changes facilitate the infant’s capacity for face-to-face interaction (Haith et al., 1977; Hopkins et al., 1990). Infants increase smiling and non-distress vocalizations (Trevarthen, 1979; Wolff, 1987). Infants also become more aware and interested in social partners (Rochat, 2001) and more responsive in interactions (Henning et al., 2005; Bigelow and Power, 2014; Beebe et al., 2016).

Yet even prior to 2 months, there is evidence of coordination between mothers and infants. For example, Murray et al. (2016) examined mother–infant interactions weekly during the infants’ first 2 months. Although minimal, infants’ social behaviors (non-distressed vocalizations, smiles) increased, particularly after 3 weeks. Mothers responded selectively to both infants’ social and non-social behaviors. Importantly, mothers’ mirroring (contingent behavior that matched the infants’ behavior) and positive responses that elicited infants’ attention (e.g., smiles, eyebrow flashes) were associated with increases in infant social behaviors.

Lavelli and Fogel (2005) examined mother–infant face-to-face interactions between birth and 3 months. Initially, infants exhibited little emotional expression. By the second month, however, they began to smile and coo and their attention became more sustained. Their behavior became linked with mothers’ responses of smiling and talking. By the end of the second month, mothers increased their “mirroring” of infant actions by matching or elaborating infant action. Turn-taking dialogs emerged with mutual attentiveness and positive affect (Lavelli and Fogel, 2013). By 2–3 months, these bi-directional sequences of positive engagement became enhanced in both partners.

Infants may be prepared to be sensitive to specific maternal responses that match or positively respond to their own behaviors, even if those responses are relatively infrequent. Infants prefer “matching” (imitative/elaborative) over non-matching forms of responses (Meltzoff, 2007; Markova and Legerstee, 2008). These preferences may involve neural mechanisms that map observed and executed expressions. Young infants may sense equivalences when their gestures are immediately observed in similar actions of others, resulting in action-perception connections that strengthen the neural circuits involved, increasing the probability of the behaviors occurring (Murray et al., 2018). Such speculation is supported by behavioral imitation studies (Simpson et al., 2014; Meltzoff and Kuhl, 2016) and neurophysiological research (Rizzolatti and Fogassi, 2014; Tramacere et al., 2016).

Mothers’ propensity to mirror (imitative/elaborative) and positively respond to certain infant behaviors over others may be a means for establishing shared communication that becomes developed and elaborated in culturally specific ways (Murray et al., 2018). More studies are needed to explore cross-cultural variations in mother–infant interactions, for example, in cultures where such interactions are less visual and more tactile (Keller, 2007; Kärtner et al., 2008, 2010; Negayama et al., 2015; Owusu-Ansah et al., 2019).

Early mother–infant interactions in non-human primates (e.g., lip smacking, mutual gaze) have been shown to affect later social–emotional functioning, suggesting an evolutionary history of early mother–infant communication patterns (Bard et al., 2005; Ferrari et al., 2009; Dettmer et al., 2016). There are, however, notable differences. In chimpanzees, these include very short durations of mutual gaze, infrequent maternal looking behavior, and the absence of such behavior after 3 months (Bard et al., 2005). Ape mothers provide caregiving and are responsive to their infants’ needs, but they rarely respond to infant vocalizations with their own or vocalize independently to their infants (Oller et al., 2019). Primary vocal intersubjectivity is virtually absent and non-vocal primary intersubjectivity is far less frequent than in humans. Overall, mother–infant interactions in non-human primates are short-lived and bear little resemblance to those observed in humans.

### Salience of Vocal Bidirectional Exchanges

Bi-directional mother–infant interactions involve all modality channels (Beebe et al., 2016). Yet by the third month, bi-directional vocal responses become particularly salient compared to bi-directional responses in facial affect (Lavelli and Fogel, 2013), at least in Western cultures where distal communication is the basis of mother–infant communication (e.g., Kärtner et al., 2010). This may be due to the ease with which infants can perceive the turn-taking quality of vocal exchanges. Mothers tend to stop talking when infants vocalize and resume talking when infant vocalization ends. Reciprocally, infants tend to become vocally responsive when mothers talk. Such interactions result in the easily recognized back and forth vocal exchanges, as first identified by Bateson (1979).

Bigelow and Power (2014) examined mother–infant face-to-face interactions at 1, 2, and 3 months and provided evidence of the primacy of vocal over facial contingency. The following patterns were observed in vocal, but not smiling exchanges. Vocal contingencies (vocal responses within 1 s of the partner’s vocalization) of mother to infant, and infant to mother, were correlated at each age. Moreover, maternal vocal contingency at 1 month predicted infant vocal contingency at 2 months, and maternal vocal contingency at 2 months predicted infant vocal contingency at 3 months. However, *infant* vocal contingency at 1 and 2 months did not predict maternal vocal contingency at 2 and 3 months, respectively. Thus, for vocal exchanges, the mother leads or scaffolds the development of contingency processes across the first 3 months.

At the end of the third month, infant vocalizations take on a new, more speech-like quality in that they are less nasalized and more fully resonant (Bloom et al., 1987; Goldstein and Schwade, 2008). Adults perceive these vocalizations as more communicative (Beaumont and Bloom, 1993; Hsu and Fogel, 2003) and respond by adjusting their own emotional responses.

Infants participate in a basic dialogic vocal turn-taking structure. Jaffe et al. (2001) investigated those dialogs by examining vocal timing coordination during mother–infant and stranger–infant face-to-face interactions in 4-month-old infants. The focus was the coordination of vocalizations, pauses, and switching pauses at the point of the turn exchange; and in particular, vocal turn-taking through the contingent coordination of switching pause durations.

**Figure 4:**
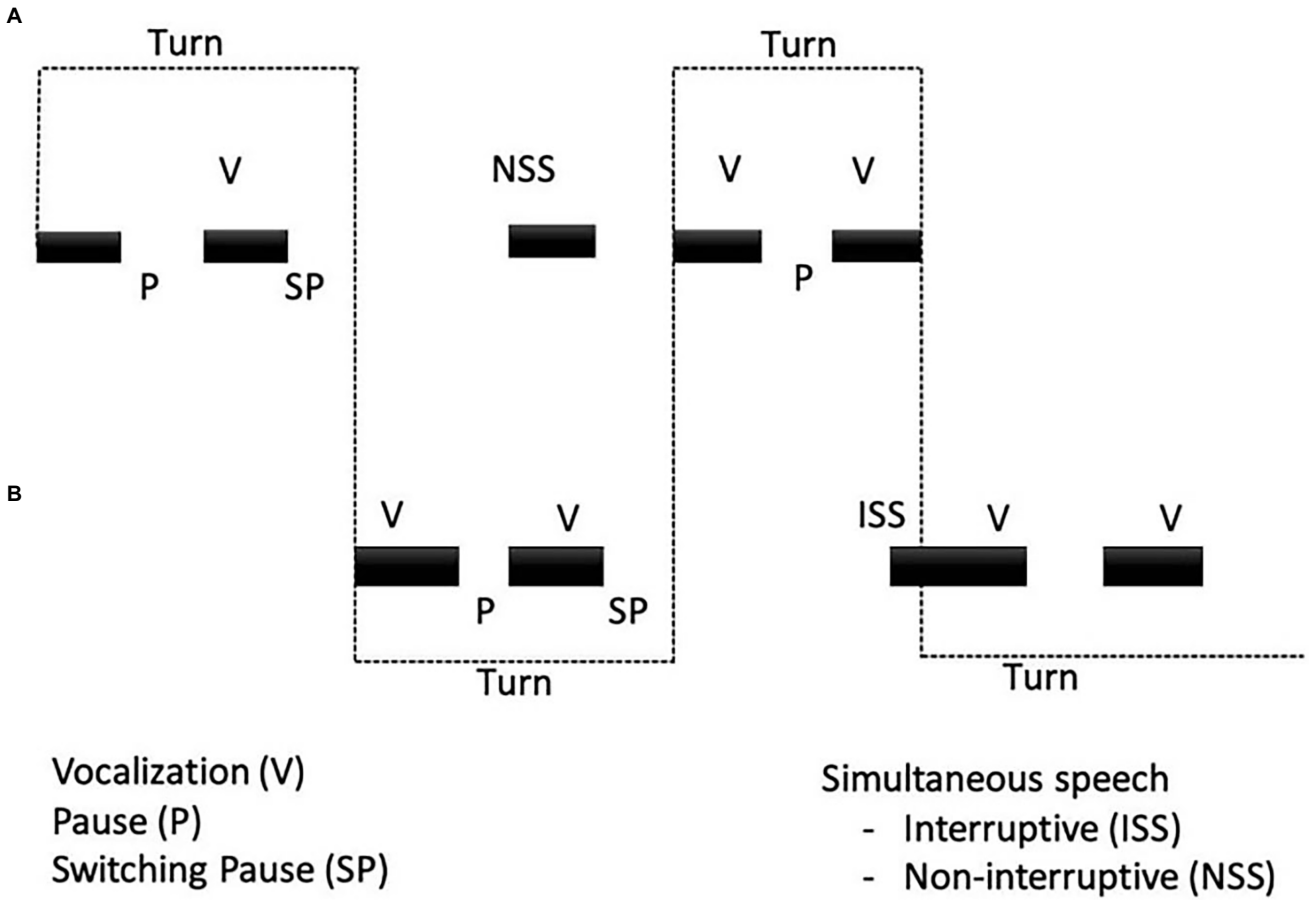
**(A)** and **(B)** represent two partners. The individual gains the turn the instant she/he vocalizes unilaterally. The switching pause (SP), which occurs as turns are exchanged, regulates the timing of turn-taking. Courtesy of Beatrice Beebe, New York State Psychiatric Institute, Columbia University.

As illustrated in Figure 4, a turn begins when either participant vocalizes alone, and it is held until the other vocalizes alone, at which point the turn is exchanged. Switching pauses occur at the moment of the turn exchange.

Infants were active participants in bi-directional contingent coordination of vocalization, with both mother and stranger. In both mother–infant and stranger–infant interactions, partners coordinated vocal turn-taking rhythms by matching the durations of “switching pauses” at the moment of the turn exchange. That is, each partner paused for similar durations before the other took a turn.

Jaffe et al. (2001) also showed that mother–infant and stranger–infant vocal timing coordination predicted outcomes during secondary intersubjectivity, specifically, 12-month attachment and infant cognition (as measured by the Bayley Scales). Infant contingent coordination was as important as adult contingent coordination in predicting outcomes, a demonstration of the infant’s role in development. Although the prediction of the Bayley Scales, a general cognitive measure, is not specific to the development of words, words develop in the context of a more general cognitive capacity.

Bornstein et al. (2015) expanded findings of Jaffe et al. (2001) in infants who were 5.5 months by documenting that dyadic conversational turn-taking exists in multiple cultures. Despite large differences in overall talkativeness of mothers and infants across the cultures, mothers’ vocalizations to their infants were contingent on the offset of infants’ non-distress vocalizations (within 2 s). Infants’ vocalizations to mothers were likewise contingent on the offset of mothers’ vocalizations.

Our expanded description of the domain of primary intersubjectivity shows that contingent coordination between mother and infant begins in the first months of life. Mothers scaffold the contingent process, but infants actively participate, and the process is bi-directional, although asymmetrical. Vocal (vs. facial) contingency is salient in this process.

## Maternal Contributions in Early Mother–Infant Interactions That Lay the Foundation for Joint Attention, and Ultimately for Words

Vygotsky (1978) and Trevarthen (1979) argued that maternal responsiveness in early interactions with infants is crucial for the development of an infant’s capacity to engage in joint attention. Although there is relatively little evidence from studies of infants under 9 months, two research groups predicted joint attention from early mother–infant interactions.

Legerstee et al. (2007) studied mother–infant face-to-face interaction in young infants, in relation to infant joint attention at 10 months. At 3 months, infant gaze and maternal attunement were measured. Maternal attunement was defined as the degree to which mothers maintained attention and displayed positive affect. At 5, 7, and 10 months, mother–infant play was examined for coordinated joint attention. Measures included the extent to which infants and mothers attended to the same object and infants shifted gaze between the mother’s face and the object. Infant gaze at 3 months predicted infant coordinated joint attention at 10 months, but only if maternal attunement was high at 3 months. These findings suggest that sensitive maternal attunement is a mechanism that fosters the link between dyadic and triadic interactions, that is, between primary and secondary intersubjectivity.

Bigelow et al. (2010) showed that mothers who were vocally contingent (within 1 s) to their infants’ vocalizations during face-to-face interactions at 4 months provided more scaffolding of infants’ joint attention (verbal encouragement, modeling, and turn-taking) at 15 months. Joint attention was defined as mothers and infants engaging with the same object and infants demonstrating awareness of the mother’s involvement through gaze, gesture, or vocalization. These results support the importance of early maternal contingent responses during primary intersubjectivity for later triadic interactions.

Although the studies cited above predicted facilitation of infant joint attention from maternal behaviors during early interactions, there have been few investigations of how such maternal behaviors might directly predict infant word acquisition or later language skills. In one of the few relevant studies, Ruddy and Bornstein (1982) found that mothers who encouraged their infants’ attention to objects during mother–infant play at 4 months had infants with larger speaking vocabularies at 12 months.

More recently, some studies have examined the relation between maternal behaviors during early mother–infant interaction and more long-term language abilities in children. Sheinkopf et al. (2017) found that mothers’ positive affect (smiling, laughter, making playful faces) and infant-directed vocalizations during face-to-face interactions with 4-month-old infants predicted the children’s verbal IQ at 4.5 years (Wechsler Preschool and Primary Scales of Intelligence, Revised) and 7 years (Wechsler Intelligence Scale for Children, 3rd edition). Bornstein et al. (2020) found that maternal language to infants (amount and frequency) and maternal sensitivity (Ainsworth Maternal Sensitivity Scale, Maternal Behavioral Q-Sort) at 5 months each independently predicted core child language skills at 49 months.

Despite the dearth of studies of maternal behavior in early infancy that predict the emergence of words, maternal behaviors in early mother–infant interaction that facilitate later infant joint attention abilities can be inferred to enhance infant word acquisition. That is because the production of words is built upon the ability to engage in joint attention (Conboy et al., 2015). Infant joint attention interactions with mother predict subsequent vocabulary size and the age at which infants begin to use words (Tamis-LeMonda et al., 1996; Carpenter et al., 1998).

Mothers’ speech to infants within joint attention is particularly facilitative of infant word learning if the mother’s speech contingently follows (rather than redirects) the infant’s object focus (Tomasello and Farrar, 1986; Akhtar et al., 1991; Dunham et al., 1993; Bigelow et al., 2010). Mothers tend to name objects that are in the center of the infant’s visual field, thereby capitalizing on the infant’s focus of attention and enhancing word learning (Yu and Smith, 2012; Pereira et al., 2014; Suanda et al., 2018). Thus, maternal speech contingent on infants’ focus during joint attention may affect infant later word learning just as maternal contingent responsiveness within early face-to-face interaction affects later infant joint attention abilities (Legerstee et al., 2007; Bigelow et al., 2010). These findings support the argument for a continuity between early prelinguistic mother–infant interaction and infants’ later emerging words. By contingently following the infant’s lead in both primary and secondary intersubjectivity, mothers facilitate their infants’ communicative abilities.

## Infant Contributions in Early Mother–Infant Interactions That Lead to Joint Attention

What do we know about the nature of infant participation in early social interactions that might be relevant to infant capacity to participate in joint attention? Direct empirical evidence is scarce. As noted previously, however, Legerstee et al. (2007) found that infant gazing at their mothers (who provided high maternal attunement) predicted infants’ later joint attention. In a rare study that documented the relation between infants’ early social behaviors and their joint attention abilities in the second year, Salley et al. (2016) found that 4-month-old infants’ social engagement with mothers in face-to-face interactions (proportion of time spent smiling, vocalizing, gazing) was associated with more frequent infant initiation of joint attention at 18 months. Infants’ early social engagement behaviors are acquired in interactions with their mothers. These encounters generate infant expectations that their actions can affect the partner’s behavior and thus enhance their sense of agency. Such abilities are crucial for later joint attention when infants engage and direct their partner’s focus to objects of their own interest.

From the beginning of infants’ increased interest in social interactions at 2 months, infants show a preference for the contingency levels they experience with their mothers. Bigelow and Rochat (2006) observed mothers and their 2-month-old infants who came to the lab in pairs. The infants engaged in face-to-face interactions with their mothers and with a stranger (mother of the other infant). Infants were most contingently responsive (smiled or vocalized within 1 s of the partner’s smile or vocalization) to the stranger if the stranger’s level of contingency to the infant was similar to that of the mother. Infants were less responsive to the stranger if the stranger’s level of contingent responsiveness differed from that of the mother. Infants’ preference for the contingency levels with which they are most familiar becomes even stronger by 4 months (Bigelow, 1998), showing infants’ growing expectation for how their partner should respond. These findings support infants’ preference for familiar contingency levels and their expectations for how interactions should unfold.

Infants’ sense of agency in affecting their partner’s behavior is apparent in the still-face phase of the Still Face Task when they demonstrate *social bids*. Social bids are smiles or non-distress vocalizations while looking at the unresponsive partner during the still-face phase. Tronick et al. (1978) were the first to suggest that these infant behaviors were efforts to elicit interaction with the unresponsive partner. Researchers have subsequently interpreted such behavior as social bids to re-engage the partner (Cohn et al., 1991; Delgado et al., 2002; Carter et al., 2008; Bigelow and Walden, 2009; Goldstein et al., 2009; Mcquaid et al., 2009; Franklin et al., 2014).

Infant social bidding behavior during the still-face phase is considered an example of infant independent initiative because social bids occur in the absence of the partner’s social behavior. Social bids not only imply that infants are aware of the effects of their own behavior, but also that infants can initiate attempts to change the partner’s behavior to repair the disrupted interaction. These are abilities that are important for joint attention, for in joint attention the infant can initiate the partner’s engagement with objects as well as shift the partner’s attention to objects that interest the infant.

Infant social bidding during the Still Face Paradigm is influenced by the degree of maternal contingency they previously experienced. In a longitudinal study with 1-, 2-, and 3-month-old infants, Bigelow and Power (2016) found that greater maternal vocal contingency in the baseline interactive phase of the Still Face Task at 2 and 3 months predicted greater likelihood of infant social bids to the mother in the still-face phase at 2 and 3 months, respectively. Moreover, maternal vocal contingency in the previous month (months 1 and 2) predicted infant social bids during the still-face phase at 2 and 3 months.

These findings illustrate the importance of an expanded view of primary intersubjectivity. The nature of maternal contingent coordination, beginning at birth, facilitates the development of the infant’s sense of agency, the expectation of the ability to affect the partner. This sense of agency will be crucial during joint attention when infants attempt to influence the partner to join their own focus of attention.

Bigelow and Power (2016) investigated the effects of both maternal vocal and smiling contingency on infant social bids. Maternal smiling contingency was not as conducive to infant social bidding as maternal vocal contingency. However, when examining older infants at 4–5 months, Mcquaid et al. (2009) showed that maternal contingent smiling to infant smiles (within 1 s) in the baseline interactive phase of the Still Face Task predicted infant smiling social bids in the still-face phase. Maternal vocal contingency was not examined in this study.

Similarly, in another Still Face study with 5-month-old infants, Bigelow et al. (2017) found that maternal contingent mirroring (within-modality or cross-modal matching of infant behavior within 1 s with vocalization, facial expression, or gesture) was associated with infant social bidding during the still-face. Infants who experienced high maternal mirroring in the interactive phases showed greater infant social bidding in the still-face phase. These studies indicate that maternal contingent behaviors make significant contributions to infants’ developing sense of agency.

Importantly, exploration of *infant contingency* (infants’ contingent responses to maternal behaviors) in predicting social bids is lacking. The one exception is Mcquaid et al. (2009), who found that infant contingent smiling to mothers’ smiles in the initial interactive phase was unrelated to infant smiling social bids in the still-face phase. A more thorough examination of the relation between infants’ contingent responsiveness and their social bidding in the absence of maternal behavior awaits future research.

That social bidding, demonstrating infant agency, is relevant to infant capacities in joint attention was shown in the Striano and Rochat (1999) study with older infants (7 and 10 months). More infant social bidding in the still-face phase predicted greater competence in triadic joint engagement tasks. These results show that infant dyadic social initiative and triadic capacities are related. Striano and Rochat (1999, p. 560) note that their results imply “a somewhat more gradual process of social cognitive developments than that implied by a suddenly emerging ‘9-month revolution,’” which is favored by Tomasello (1999).

## Future Research Directions for Exploring the Continuity of Primary and Secondary Intersubjectivity

We have argued that there is continuity between primary and secondary intersubjectivity and that both are necessary for the emergence of words. Infants share a full range of attention and emotion with their caregivers dyadically during their early months. Toward the end of the first year, they share attention triadically to objects in their immediate environment, which culminates in word acquisition.

To be sure, the cognitive and social requirements for word learning go beyond the achievements of intersubjectivity. Infants’ further development of symbolic capacity, of which words are only one example, are also necessary as are neural and motor developments (Deacon, 1997). Everett (2017) describes various cognitive and cultural influences that make language possible.

Although research tracking the continuity of primary and secondary intersubjectivity is impressive, there are important gaps in the literature that should be addressed. First, longitudinal research is needed that follows early infant–adult interactions from primary intersubjectivity through to secondary intersubjectivity and ultimately to the acquisition of words. That research should evaluate how maternal behavior in early dyadic interactions with infants influences the subsequent development of joint attention. Importantly, studies documenting the role of infants in this development from primary to secondary intersubjectivity are sorely needed. Studies of the infant’s role in the continuum from dyadic to triadic interactions, or the infant capacities necessary for triadic interactions, are scarce. Studies that examine how infant behaviors in early face-to-face interactions affect their later joint attention behaviors should be the focus of future work.

Second, most of the studies inferring the continuity of primary and secondary intersubjectivity have been correlational. Although longitudinal studies show associations between early maternal contingent behavior and later infant joint attention behaviors (Legerstee et al., 2007; Bigelow et al., 2010), experimental studies are needed. Such studies are likely to be intervention studies or studies that include infants with impairments of key abilities important to intersubjectivity, for example, infants with perceptual deficits, such as blindness or deafness (e.g., Bigelow, 2003; Depowski et al., 2015) or autistic children in whom the ability to engage with others is compromised (Cassel et al., 2007; Wan et al., 2013).

Third, more cross-cultural studies on intersubjectivity are needed. Most of the studies concerning intersubjectivity have been conducted in Western societies, where distal parenting practices focus on face-to-face interactions and object play. However, many non-Western societies have proximal parenting practices that emphasize physical contact and body stimulation. Some cross-cultural studies show that maternal responsiveness is similar in distal and proximal parenting cultures, although manifested differently (Keller et al., 2004; Keller, 2007; Kärtner et al., 2008, 2010). Mothers in distal parenting cultures are more likely to be verbally responsive to their infants, whereas mothers in proximal parenting cultures tend to use physical contact responses.

Interestingly, the mode of maternal responsiveness between distal and proximal parenting cultures diverges around the infant age of 2 months (Kärtner et al., 2008, 2010), when infants’ perceptual-motor abilities increase their capacities for social engagement. Mothers from distal parenting cultures tend to reduce tactile responses to infants between 2 and 3 months and increase face-to-face interactions with facial and vocal responses, whereas mothers in proximal parenting cultures tend to continue to use high levels of tactile responsiveness (Kärtner et al., 2008, 2010). Although infant biological maturation is universal and infants are predisposed to engage with others, biological predispositions interact with parenting practices early in life and adapt to cultural demands. Thus, we need research on how culture affects infant development from primary to secondary intersubjectivity.

## Why the Emergence of Words Is Unique in Humans

The title of this article, “Intersubjectivity and the Emergence of Words,” implies that words are well defined. Remarkably, psychologists and linguists have yet to agree about a definition of a word. In fact, that issue has rarely been considered.

The absence of a clear definition has led to many ambiguities about the type of utterances that count as words. Chomsky, for example, thinks that origin of words is a mystery: “The minimal meaning-bearing elements of human languages…are radically different from anything known in animal communication systems. Their origin is entirely obscure, posing a serious problem for the evolution of human cognitive capacities, particularly language” (Berwick and Chomsky, 2016, p. 90–91).

Some scholars have argued that words are not uniquely human. In a widely cited article, Hauser et al. (2002) distinguished two “faculties of language”: a broad faculty that includes, among other abilities, words and concepts, and a narrow faculty that includes grammar. In that framework, they concluded that only the narrow faculty is uniquely human.

We agree that the use of grammar is uniquely human. But here, we define words in a way that warrants their inclusion in the narrow faculty of language, a faculty that is uniquely human. We define words functionally, as arbitrary symbols that are used conversationally, that is, declaratively. Their function is to transmit information socially by referring to particular objects, activities, or their attributes. Later in development, words can also refer to internal states. This definition implies that only humans use words. It also recognizes the social origins of words.

Our definition of a word differs from that of many scholars who study the communicative abilities of animals. As evidence that animals use words, they cite the communicative abilities of chimpanzees, monkeys, dolphins, dogs, and birds (Savage-Rumbaugh et al., 1993; Hauser et al., 2002; Kaminski et al., 2004; Seyfarth et al., 2005; Pepperberg, 2016). It is important to note that none of those studies defined words.

Another problem is the distinction between comprehension and production. Studies of comprehension cannot provide a definitive answer to the question of whether animals use words because it is not clear if a subject’s response to an experimenter’s vocal command is based on the perception of its acoustic properties or its lexical status. That problem arises both in instances of individual commands (e.g., dogs, Kaminski et al., 2004) and in sequences of words (e.g., chimpanzees, Savage-Rumbaugh et al., 1993).

Studies of production often fail to distinguish between declarative and imperative functions of communication. Regarding chimpanzees, Berwick and Chomsky (2016, p. 148) cited the ability of Nim, a chimpanzee trained by Terrace et al. (1979) to produce words. It is true that apes can be trained to use sign language or arbitrary visual symbols to communicate (Gardner and Gardner, 1969; Premack, 1971; Rumbaugh, 1977; Terrace et al., 1979; Savage-Rumbaugh, 1994). In criticizing claims that those studies provide evidence that apes use words, however, Terrace (2019) argued that the responses in question only served an imperative function of obtaining specific rewards.

Imperatives are responses to satisfy a need, whereas declaratives are responses that refer to objects in a conversational manner. The following example illustrates the difference between utterances of apes and humans: an imperative in the case of the former, a declarative in the case of the latter. Having been shown a dog or a picture of a dog, the ape might sign *dog*, or touch a lexigram meaning *dog*, in order to obtain food or drink. The sight of a dog was simply a cue for making a response to obtain a physical reward. By contrast, if an infant sees a dog or a picture of a dog, she might utter *dog*, in response to which her caretaker responds socially, typically, with other words, for example, *nice dog*, *big dog*, *no that’s a cat*, and so on.

In discussing differences between the utterances of apes and humans, Terrace (1985) noted that the utterances of human infants are spontaneous and bi-directional, whereas ape utterances are neither. Most important is an ape’s inability to name or refer to objects in a declarative way.

In humans, utterances that produce primary rewards (imperatives), like a morsel of food, make up a miniscule portion of their vocabulary. If, as with apes, such utterances were the only ones a human could learn, language would never develop. From the beginning of word acquisition, the vast majority of human utterances are declaratives.

In any of the thousands of extant human languages, the number of declarative words is unlimited. It is always possible to conceive of a new word to name a particular object, action, or attribute. It is that feature that allowed our ancestors to refer to objects that were not immediately present, to past and future events, and to imaginary objects. In short, the transition from animal communication to declarative words marked the beginning of verbal culture. That transition took place because of the development of intersubjectivity.

## Evolution of Intersubjectivity

From birth, infants embark on a trajectory of primary and secondary intersubjective engagements with their caretakers that are uniquely human. How did such interpersonal relations evolve? In particular, from what aspects of our ancestors’ behavior did a high degree of social coordination and cooperation, both crucial features of intersubjectivity, evolve? To answer that question, we need to identify the selection pressures that favored increases in social communication and intention-reading.

Looking at chimpanzees, our closest living ancestors, infant–mother relations differ profoundly from those of humans. Although some features of intersubjectivity, for example, mutual eye gaze, have been observed in chimpanzees, they are short-lived and disappear when infants are a few weeks old (Bard, 2011). As noted by Oller et al. (2019), ape mothers “do not respond to infant vocalizations with vocalizations of their own, and rarely if ever vocalize independently to their infants.”

According to Hrdy (2009), the evolutionary origins of intersubjectivity can be found in the difference in child-rearing practices in apes and humans. Chimpanzee mothers do not allow other members of their group access to their infants for approximately 6 months. For gorillas and orangutans, that period is longer.

By contrast, human infants are reared by cooperative child-rearing, a practice in which a mother’s care of her infant is supplemented by members of her immediate family, so-called “alloparents.” The mother is still the primary source of care but sisters, brothers, aunts, fathers, and grandmothers, even non-kin, also share in caring for newborn human infants.

To survive, infants have to rely not only on their mothers, but also on their alloparents. Thus, human infants have to learn to assess the emotions and intentions of alloparents, as well as those of the mother. They begin to do that right after birth. By contrast, infant apes rely only on their mothers.

There is compelling evidence that cooperative child-rearing was practiced by *Homo erectus*, a human ancestor who evolved about 1.8 million years ago (O’Connell et al., 1999). It is likely that *Homo erectus* infants, and their multiple caregivers, were socially involved in ways that apes never were. *Homo erectus* infants had to learn to interpret not only their mothers’ engagement but also the moods and intentions of alloparents who might help.

How best to attract care under such circumstances? Hrdy (2009) argues by engaging socially with a caregiver, by crying, smiling, vocalizing, or gesturing. Those infants who were best at engaging in the non-verbal communication that defines intersubjectivity would be the best cared for. Such novel selection pressures favor a very different type of ancestor, one that Hrdy refers to as “emotionally modern.” They were, as Hobson (2002) noted in the epigraph, mothers and alloparents who could share attention and emotions with their infants, and infants who could reciprocally communicate their attention and emotions.

Hrdy also notes that human ancestors were emotionally modern before they became anatomically or cognitively modern: “Long before the emergence of *anatomically modern* big-brained humans…, or before…symbolic thought and language, these emotionally different apes [actually *Homo erectus*] were already eager to appeal to and help others” (Hrdy and Burkart, 2020, p. 8, italics in original).

Recent research also suggests the altricial nature of *Homo erectus*. The birth canal of *Homo erectus* had narrowed to the point at which, like humans, the size of an infant’s brain at birth was relatively small (Simpson et al., 2008; Gruss and Schmitt, 2015). That suggests that, like modern human infants, newborn *Homo erectus* infants required long-term caretaking in order to survive, thus characterizing them as altricial.

Locke and Bogin (2006) and Oller and Griebel (2021) hypothesized that, as a result of their altricial needs, there was intense pressure for *Homo erectus* infants to provide fitness signals to their caregivers for long-term nurturance and protection. Specifically, they hypothesized that vocalization, expressed as protophones, satisfied that pressure. Oller and Griebel (2021, p. 8) conjectured that “relative altriciality and cooperative breeding may have co-evolved, with both supplying selective pressure and vocal fitness signaling in the hominin [Homo] case.”

### Hrdy’s and Tomasello’s Views of the Evolution of Intersubjectivity

In this context, it is important to note differences between Hrdy’s and Tomasello’s approaches to intersubjectivity. Tomasello argues that the cognitive differences between chimpanzees and humans stem from the type of tasks on which those differences are evaluated. When the task is competitive, chimpanzees are able to read another’s intentional stance as well as humans. It is only in cooperative tasks in which chimpanzees and humans differ.

In contrast to Tomasello, and in agreement with Hrdy, we would argue that the difference is more fundamental. The competitive task obscures the actual difference because it does not take into account differences in intersubjectivity in humans and chimpanzees. The chimpanzee’s ability to read another’s intentional stance differs from the human’s ability to share intentions and communicate about them in a bi-directional fashion.

Moreover, Tomasello did not specify the origins of a high degree of social coordination and cooperation in humans. In his “interdependence hypothesis,” Tomasello et al. (2012) maintained that shared intentionality in humans is an adaptation mainly for *adults*’ uniquely cooperative forms of social life. Only recently, however, did Tomasello acknowledge Hrdy’s view that cooperative breeding was key in an infant’s ability to solicit care and attention and to develop shared intentionality (Tomasello and Gonzalez-Cabrera, 2017; Tomasello, 2020).

## Conclusion

Beginning with emotionally modern ancestors, in whom it is likely that intersubjectivity first developed, there was a remarkable transition in communication. The shift from a limited number of uni-directional emotional signals, which many animals share with humans, to intersubjectivity, was a shift to bi-directional, moment-by-moment emotional and cognitive communication that starts at birth. Such reciprocally contingent communication is crucial for the emergence of words.

Research on interactions in *early infancy*, particularly the key role of contingency in mother–infant prelinguistic communication, shows that an infant’s progress toward joint attention and word learning, rather than being a product of a 9-month revolution, begins at birth and is an incremental process of infant social development to which both mother and infant contribute.

Early bi-directional communication between infant and caregiver is facilitated by maternal scaffolding of infant communicative abilities. It culminates with joint attention and the emergence of words, which ultimately generates an indeterminately large number of voluntary and arbitrary symbols. That is the basis for grammar, a complex topic that lies outside the scope this article.

The evolution of words could not have occurred without primary intersubjectivity. The emotional communication that an infant experiences with her caregiver from the beginning of life is foundational for the emergence of words.

## Author Contributions

HT, AB, and BB contributed equally to the writing of the article and approved the submitted version.

## Funding

This study was partially funded by grants to HT (NIMH MH 111703), to BB (NIMH MH 56130, the Bernard and Esther Besner Infant Research Fund, the Hispanic Federation and the Köhler Foundation), and to AB (Nova Scotia Health Research Foundation, PSO-Project-2003-350, and the Natural Sciences and Engineering Research Council of Canada, RGPIN-2016-03936).

## Conflict of Interest

The authors declare that the research was conducted in the absence of any commercial or financial relationships that could be construed as a potential conflict of interest.

## Publisher’s Note

All claims expressed in this article are solely those of the authors and do not necessarily represent those of their affiliated organizations, or those of the publisher, the editors and the reviewers. Any product that may be evaluated in this article, or claim that may be made by its manufacturer, is not guaranteed or endorsed by the publisher.
